# NanoPDLIM2-Based Combination Therapy for Lung Cancer Treatment in Mouse Preclinical Studies

**DOI:** 10.21769/BioProtoc.5437

**Published:** 2025-09-05

**Authors:** Thi Hoa Le, Fan Sun, Gutian Xiao, Zhaoxia Qu

**Affiliations:** 1 Norris Comprehensive Cancer Center, Hastings Center for Pulmonary Research, Department of Molecular Microbiology and Immunology, University of Southern California Keck School of Medicine, Los Angeles, CA, USA; 2 UPMC Hillman Cancer Center, Department of Microbiology and Molecular Genetics, University of Pittsburgh School of Medicine, Pittsburgh, PA, USA

**Keywords:** NanoPDLIM2, Lung cancer, PD-1 blockade, Chemotherapy, Immunotherapy, Nanoparticle delivery, Immune checkpoint inhibitors, Combination therapy

## Abstract

This protocol describes the preparation, administration, and analysis of a nanoparticle-based therapeutic strategy (nanoPDLIM2) in combination with PD-1 immune checkpoint blockade immunotherapy and chemotherapy for the treatment of lung cancer in mouse preclinical studies. NanoPDLIM2 uses a polyethyleneimine (PEI)-based delivery system that encapsulates PDLIM2 expression plasmids for reconstituting PDLIM2 that is repressed in tumors. This approach induces tumor immunogenicity, suppresses drug resistance, and improves treatment efficacy when used in combination with carboplatin, paclitaxel, and anti-PD-1 antibodies. The protocol describes steps for mouse lung tumor induction, nanoPDLIM2 and other therapeutic reagents’ preparation and administration, and subsequent analysis of tumor burden, immune response, and toxicity, providing a reproducible approach for investigators.

Key features

• Comprehensive workflow for preparation and delivery of nanoPDLIM2.

• Combination of nanoPDLIM2 with PD-1 blockade and chemotherapeutics for superior efficacy in lung cancer treatment.

• Detailed protocols for therapeutic reagents preparation, administration, tumor examination, immune analysis, health monitoring, and toxicity evaluation in a preclinical lung cancer model.

## Graphical overview


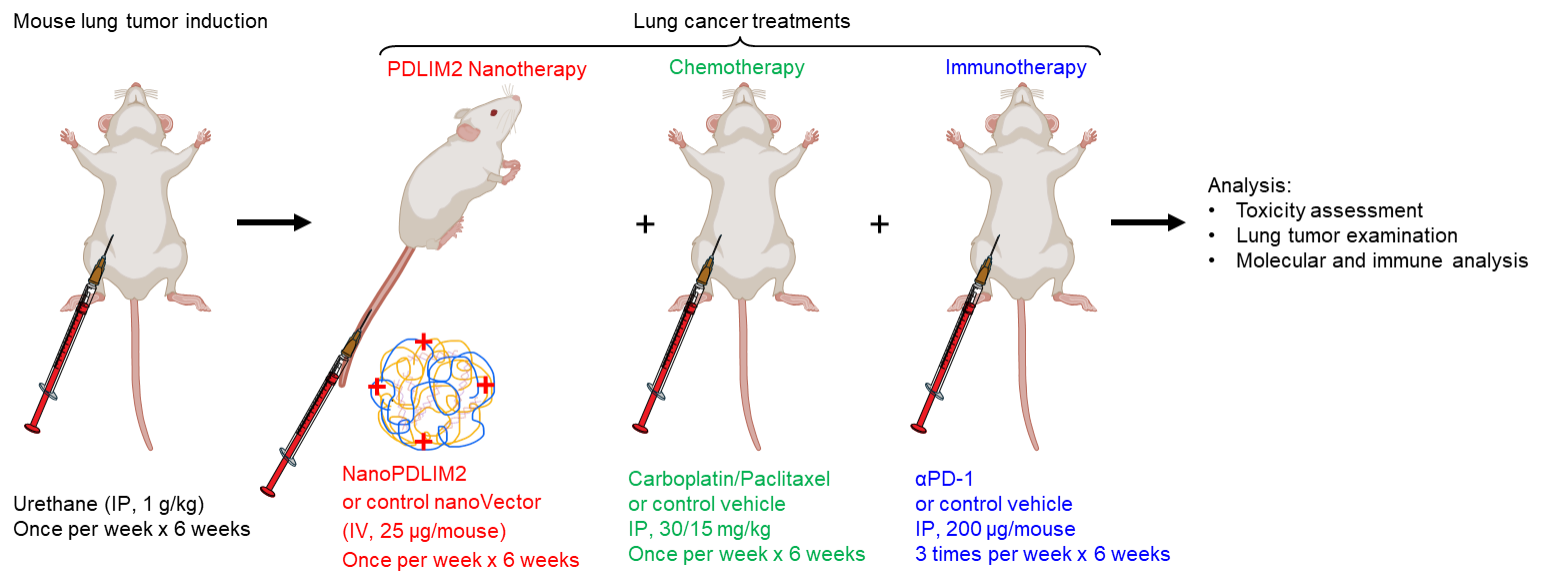


## Background

Lung cancer remains the leading cause of cancer-related mortality worldwide, affecting both men and women. Despite advances in therapy, the 5-year survival rate remains low at only 25% [1]. In recent years, immune checkpoint blockade immunotherapy, particularly targeting PD-1/PD-L1, has become a pivotal strategy for treating lung cancer, including non-small-cell lung cancer (NSCLC) and small-cell lung cancer (SCLC). However, the response rate to PD-1/PD-L1 blockade remains limited, with only about 20% of lung cancer patients showing meaningful clinical benefits [2]. This limited efficacy is largely attributed to the immunologically "cold" nature of most lung tumors, characterized by sparse T-cell infiltration, low immunogenicity, and minimal PD-L1 expression [3,4].

Combination of immune checkpoint blockade immunotherapy with other therapies has been actively pursued to improve treatment outcomes [4–8]. Chemotherapy has been shown to increase tumor immunogenicity and PD-L1 expression, offering a rationale for combining it with immune checkpoint inhibitors (ICIs) to improve treatment outcomes. Indeed, such combination therapies have improved response rates and prolonged survival in preclinical and clinical studies [5,6,8]. Nonetheless, even combined approaches rarely achieve complete tumor regression, and progression-free survival remains limited. A major barrier to treatment efficacy is tumor-driven immune evasion via downregulation of MHC class I, which is critical for CD8^+^ T-cell-mediated recognition [8–11].

PDLIM2, a PDZ-LIM domain-containing protein, is a tumor suppressor implicated in the pathogenesis of various cancers, and particularly, lung cancer [8–18]. One key mechanism underlying PDLIM2’s tumor-suppressive effects involves the negative regulation of the nuclear factor kappa B (NF-κB) and signal transducer and activator of transcription 3 (STAT3) signaling pathways [8–15,19–21]. NF-κB and STAT3 are two master transcription factors that play a central role in inflammation, metabolic reprogramming, and cell survival, and are thereby highly pro-tumorigenic [22–38]. A recent mechanistic study suggests that instead of being claimed before as a ubiquitin ligase (E3), PDLIM2 acts as a novel ubiquitin ligase enhancer (E5) to stabilize and chaperone the E3 SCF^β-TrCP^ to ubiquitinate nuclear RelA for proteasomal degradation, therefore suppressing NF-κB activity [39,40]. RelA, also known as p65, is the prototypical member of NF-κB that is persistently activated in lung and many other cancers [22–27]. PDLIM2 is ubiquitously expressed under physiological conditions, with the highest level in the lung and lung epithelial cells in particular [9–11,41–43]. Notably, PDLIM2 is frequently repressed in lung tumors through both genetic loss and epigenetic silencing, with loss of heterozygosity and/or promoter methylation observed in over 90% of lung cancer patients [10]. More importantly, loss of PDLIM2 in lung tumors contributes to immune evasion, reduced MHC-I expression, increased chemoresistance, and activation of survival pathways, including upregulation of multidrug resistance genes like MDR1, promoting lung cancer and its therapy resistance [8–11].

To overcome the barriers to lung cancer treatment efficacy, a novel nanotechnology-based therapeutic strategy—nanoPDLIM2—has been developed to restore PDLIM2 expression in tumors. This approach uses systemically delivered nanoparticles containing PDLIM2-expressing plasmids to re-establish tumor suppressive and immune-stimulating functions. When combined with chemotherapy and PD-1 blockade, nanoPDLIM2 treatment resulted in robust tumor eradication in preclinical lung cancer models without added toxicity [10].

The following experimental protocol describes the procedures and analysis of the triple-combination treatment—nanoPDLIM2, chemotherapy, and PD-1 immune checkpoint blockade—in urethane-induced lung tumors in mice, a murine lung cancer model that recapitulates human lung cancer in many aspects, including histology, genetics, molecular biology, and immunology [7,8,10,21,26–28]. Urethane is a chemical carcinogen present in fermented food, alcoholic beverages, and cigarette smoke, the predominant risk factor accounting for about 90% of human lung cancer cases [44]. Like in human lung tumors, PDLIM2 is also repressed in urethane-induced murine lung tumors [8–10]. This protocol provides a reproducible platform to investigate the translational potential of restoring PDLIM2 expression to overcome therapeutic resistance and enhance immune responses in lung cancer. This NanoPDLIM2-based combination therapy may be effective for treating other cancers, since PDLIM2 repression has also been linked to numerous human cancers other than lung cancer [8–18].

## Materials and reagents

**Biological materials**

1. FVB/NJ mice (an inbred strain with normal lifespan under lab conditions) (The Jackson Laboratory, catalog number: 001800)

**Reagents**

1. Sodium chloride (NaCl) (Fisher Scientific, catalog number: BP358)

2. Potassium chloride (KCl) (Fisher Scientific, catalog number: BP3661)

3. Sodium phosphate dibasic heptahydrate (Na_2_HPO_4_·7H_2_O) (MilliporeSigma, catalog number: S9390)

4. Potassium phosphate monobasic (KH_2_PO_4_) (MilliporeSigma, catalog number: P5655)

5. Urethane (MilliporeSigma, catalog number: U2500)

6. PDLIM2 expression plasmid pCMV-myc-PDLIM2 (reagent from the lab)

7. Plasmid vector control pCMV (reagent from the lab)

8. Polyethylenimine (in vivo-jetPEI^®^, Polyplus Transfection, catalog number: 101000030, includes 10% glucose)

9. Saline (0.9% sodium chloride for injection) (Medline, catalog number: ABB048881010Z)

10. Carboplatin (AdipoGen, catalog number: AG-CR1-3591)

11. Paclitaxel (AdipoGen, catalog number: AG-CN2-0045)

12. EndoFree Plasmid Maxi kit (Qiagen, catalog number: 12362)

13. LB broth, Miller Luria broth (MilliporeSigma, catalog number: 717535)

14. LB broth with agar (Miller, MilliporeSigma, catalog number: L3147)

15. Ampicillin sodium salt (BioXtra, MilliporeSigma, catalog number: A8351)

16. Competent *E. coli* strain DH5α (Thermo Fisher Scientific, catalog number: 18265017)

17. Dimethyl sulfoxide (DMSO) (Fisher Scientific, catalog number: BP231)

18. Tween 80 (MilliporeSigma, catalog number: P1754)

19. Ketamine (100 mg/mL) (Henry Schein Animal Health, catalog number: 056344)

20. Xylazine (20 mg/mL) (Henry Schein Animal Health, catalog number: 033197)

21. Neutral buffered formalin (NBF), 10% formalin in PBS (Thermo Fisher Scientific, catalog number: 5705)

22. Tissue-Tek optimal cutting temperature (OCT) compound for fresh tissue embedding for frozen sections (Sakura Finetek, catalog number: 4583)

23. Collagenase D (MilliporeSigma, catalog number: 11088866001)

24. DNase I (Roche, catalog number: 10104159001)

25. Fetal bovine serum (FBS) (ATCC, catalog number: 30-2020)

26. RPMI 1640 medium with HEPES and L-glutamine (Cytiva, catalog number: SH30255.FS)

27. Penicillin-streptomycin (10,000 U/mL) (Thermo Fisher Scientific, catalog number: 15140122)

28. Ammonium chloride (NH_4_Cl) (MilliporeSigma, catalog number: 326372)

29. Potassium bicarbonate (KHCO_3_) (MilliporeSigma, catalog number: 237205)

30. Ethylenediaminetetraacetic acid disodium salt dihydrate (Na2-EDTA) (MilliporeSigma, catalog number: E4884)

31. Bovine serum albumin (BSA) (MP Biomedical, catalog number: 0219989850)

32. Intracellular (IC) fixation buffer (Thermo Fisher Scientific, catalog number: 00-8222-49)

33. 10× permeabilization buffer (Thermo Fisher Scientific, catalog number: 00-8333-56)

34. Phorbol 12-myristate 13-acetate (PMA) (MilliporeSigma, catalog number: P1585)

35. Ionomycin (MilliporeSigma, catalog number: I0634)

36. Brefeldin A solution, 1,000× (BFA, 3 mg/mL) (Thermo Fisher Scientific, catalog number: 00-4506-51)

37. Monensin solution, 1,000× (2 mM) (Thermo Fisher Scientific, catalog number: 00-4505-51)

38. Antibodies (Table 1)

**Table 1.** Antibodies used

Antibody	Company	Cat. No.	Dose	Usage
Anti-PDLIM2	Everest Biotech	EB11878	1:400	IHC
Anti-Cleaved Caspase 3	Cell Signaling Technology	9661	1:200	IHC
Anti-Bcl-xL	Cell Signaling Technology	2764	1:2,400	IHC
Anti-Cyclin D1	Santa Cruz Biotechnology	sc-450	1:200	IHC
Anti-BrdU	Sigma-Aldrich	B2531	1:500	IHC
Anti-RelA	Cell Signaling Technology	8242	1:500	IHC
Anti-STAT3	Cell Signaling Technology	4904	1:1,000	IHC
Anti-MDR1	Cell Signaling Technology	13978	1:800	IHC
Anti-CD4	Abcam	ab183685	1:1,000	IHC
Anti-CD8	Abcam	ab209775	1:1,000	IHC
Anti-PD-L1	Cell Signaling Technology	64988	1:200	IHC
Anti-MHC-I	Abcam	ab15681	1:200	IHC
Anti-mouse IgG biotinylated	Vector Laboratories	BMK-2202	1:200	IHC
Goat anti-rat IgG biotinylated	Vector Laboratories	BA9401	1:100	IHC
Rabbit anti-goat IgG biotinylated	Santa Cruz Biotechnology	sc-2774	1:200	IHC
Goat anti-rabbit IgG biotinylated	Dako	E0432	1:200	IHC
Anti-Hsp90	Santa Cruz Biotechnology	sc-13119	1:1,000	WB
Anti-c-Myc	Santa Cruz Biotechnology	sc-40	1:1,000	WB
Anti-CD16/CD32	eBioscience	14-0161-85	1.0 μL per sample	FACS
Anti-CD45 FITC	Biolegend	103107	0.5 μL per sample	FACS
Anti-EpCAM PE	eBioscience	12-5791-82	0.625 μL per sample	FACS
Anti-MHC-I APC	eBioscience	17-5998-82	1.25 μL per sample	FACS
Anti-CD3 PE	eBioscience	12-0031-83	2.5 μL per sample	FACS
Anti-CD4 PE-Cy7	eBioscience	25-0042-82	1.25 μL per sample	FACS
Anti-CD8a APC	eBioscience	17-0081-83	0.625 μL per sample	FACS
Anti-IFNγ FITC	eBioscience	11-7311-82	1 μL per sample	FACS
Anti-PD-1	BioXcell	BE0273	200 μg/mouse/time	in vivo blockade

**Solutions**

1. Phosphate-buffered saline (PBS) (see Recipes)

2. Saline (see Recipes)

3. Urethane solution (see Recipes)

4. LB medium (see Recipes)

5. Ampicillin (100 mg/mL) (see Recipes)

6. LB medium with ampicillin (see Recipes)

7. LB agar plates with ampicillin (see Recipes)

8. Anti-PD-1 antibody injection solution (see Recipes)

9. Carboplatin stock solution (see Recipes)

10. Paclitaxel stock solution (see Recipes)

11. Carboplatin injection solution (see Recipes)

12. Paclitaxel injection solution (see Recipes)

13. Plasmid DNA solution (see Recipes)

14. In vivo-jetPEI solution (see Recipes)

15. NanoPlasmid solution (see Recipes)

16. Anesthesia solution (see Recipes)

17. Collagenase D solution (see Recipes)

18. DNase I solution (see Recipes)

19. Lung digestion solution (see Recipes)

20. Red blood cell (RBC) lysis buffer (see Recipes)

21. Flow cytometry staining buffer (see Recipes)

22. Culture medium (see Recipes)

23. PMA stock solution (see Recipes)

24. Ionomycin stock solution (see Recipes)

25. 1× Permeabilization Buffer (see Recipes)

**Recipes**

**1. Phosphate-buffered saline (PBS)**

**Table:** 

Reagent	Final concentration	Quantity or Volume
KCl	2.7 mM	0.201 g
NaCl	137 mM	8.0 g
Na_2_HPO_4_·7H_2_O	10 mM	2.68 g
KH_2_PO_4_	1.8 mM	0.245 g
ddH_2_O	n/a	up to 1,000 mL

After dissolving the reagents, adjust pH to 7.2–7.4. Sterilize by autoclaving at 121 °C for 25 min.

**2. Saline**

**Table:** 

Reagent	Final concentration	Quantity or Volume
NaCl	0.9% (w/v)	9 g
ddH_2_O		1,000 mL

Sterilize by autoclaving at 121 °C for 25 min after reagent dissolution.

**3. Urethane solution**

**Table:** 

Reagent	Final concentration	Quantity or Volume
Urethane	100 mg/mL	1 g
PBS		10 mL

Dissolve 1 g of urethane in 10 mL of sterile PBS to make a 100 mg/mL solution and filter-sterilize the solution using a 0.22 μm syringe filter.

*Note: Prepare freshly the same day before each injection for best results.*

**4. LB medium**

**Table:** 

Reagent	Final concentration	Quantity or Volume
LB broth	25 g/L	25 g
ddH_2_O	n/a	up to 1,000 mL

Formulation per liter: 5 g of yeast extract, 10 g of peptone from casein, and 10 g of NaCl. To prepare 1 L of LB medium, add 25 g of LB broth to 1 L of distilled water and mix by vortexing. Adjust the pH to 7.0 with 5 N NaOH and sterilize by autoclaving for 25 min at 121 °C.

**5. Ampicillin (100 mg/mL)**

**Table:** 

Reagent	Final concentration	Quantity or Volume
Ampicillin	100 mg/mL	1 g
ddH_2_O	n/a	up to 10 mL

Dissolve 1 g of ampicillin in 10 mL of sterile ddH_2_O to make a 100 mg/mL solution and filter-sterilize the solution using a 0.22 μm syringe filter. Aliquot in 500 μL/aliquot and store at -80 °C.

**6. LB medium with ampicillin**

**Table:** 

Reagent	Final concentration	Quantity or Volume
LB medium		499.5 mL
Ampicillin (100 mg/mL)	100 μg/mL	0.5 mL
Total	-	500 mL

**7. LB agar plates with ampicillin**

**Table:** 

Reagent	Final concentration	Quantity or Volume
LB broth with agar		499.5 mL
Ampicillin (100 mg/mL)	100 μg/mL	0.5 mL
Total	n/a	500 mL

Formulation per liter: 10 g of tryptone, 5 g of yeast extract, 10 g of NaCl, 15 g of agar. To prepare 500 mL of LB agar, add 20 g of LB agar powder to 500 mL of distilled water and mix thoroughly by vortexing or stirring. Adjust the pH to 7.0 using 5 N NaOH if necessary. Sterilize by autoclaving at 121 °C for 25 min. Let the medium cool to ~50 °C before adding antibiotics and pouring them into plates. Pour around 20–25 mL of LB agar per 10 cm Petri dish plate.

**8. Anti-PD-1 antibody injection solution**

Prepare the solution in sterile tubes/vials aseptically in a biosafety cabinet. The concentration of anti-PD-1 antibody varies with different batches. Aliquot the antibody in sterile tubes based on the number of mice to be treated each time. For example, with an antibody concentration of 6.5 mg/mL, aliquot 308 μL of antibody solution for 10 mice to be treated each time (200 μg/mouse/6.5 mg/mL × 10 mice = 30.8 μL/mouse × 10 mice = 308 μL). Store at -80 °C. When it is time for injections, add sterile PBS to 100 μL per 200 μg of antibody.

**9. Carboplatin stock solution**

**Table:** 

Reagent	Final concentration	Quantity or Volume
Carboplatin	10 mg/mL	100 mg
PBS	n/a	up to 10 mL

Prepare the solution in sterile tubes/vials aseptically in a biosafety cabinet. Aliquot at 1.5 mL/aliquot in sterile tubes and store at -80 °C.

**10. Paclitaxel stock solution**

**Table:** 

Reagent	Final concentration	Quantity or Volume
Paclitaxel	30 mg/mL	100 mg
DMSO	n/a	3.33 mL

Prepare the solution in sterile tubes/vials aseptically in a biosafety cabinet. Aliquot at 250 μL/aliquot in sterile tubes and store at -80 °C.

**11. Carboplatin injection solution**

**Table:** 

Reagent	Final concentration	Quantity or Volume
Carboplatin stock solution	3 mg/mL	1.5 mL
PBS		3.5 mL
Total	n/a	5 mL

Prepare the solution in sterile tubes/vials aseptically in a biosafety cabinet. Use the same day it is prepared.

**12. Paclitaxel injection solution**

**Table:** 

Reagent	Final concentration	Quantity or Volume
Paclitaxel stock solution	1.5 mg/mL	250 μL
Tween 80	n/a	250 μL
PBS	n/a	4.5 mL
Total	n/a	5 mL

Prepare the solution in sterile tubes/vials aseptically in a biosafety cabinet. Dilute paclitaxel stock solution (30 mg/mL) with 1× volume of Tween-80 and then 18× volume of PBS to a 1.5 mg/mL final concentration. Use the same day it is prepared.

**13. Plasmid DNA solution**

**Table:** 

Reagent	Final concentration	Quantity or Volume
*Pdlim2* or control vector plasmid DNA	n/a	25 μg
10% glucose solution	5%	50 μL
Sterile water	n/a	50 μL
Total	n/a	100 μL

Prepare the solution in sterile tubes/vials aseptically in a biosafety cabinet.

**14. In vivo-jetPEI solution**

**Table:** 

Reagent	Final concentration	Quantity or Volume
In vivo-jetPEI	n/a	4 μL
10% glucose solution	5%	50 μL
Sterile water	n/a	46 μL
Total	n/a	100 μL

Prepare the solution in sterile tubes/vials aseptically in a biosafety cabinet.

**15. NanoPlasmid solution**

**Table:** 

Reagent	Final concentration	Quantity or Volume
In vivo-jetPEI solution	n/a	100 μL
Plasmid DNA solution	n/a	100 μL
Total	n/a	200 μL

Prepare the solution in sterile tubes/vials aseptically in a biosafety cabinet.

**16. Anesthesia solution**

**Table:** 

Reagent	Final concentration	Quantity or Volume
Ketamine (100 mg/mL)	9 mg/mL	900 μL
Xylazine (20 mg/mL)	1 mg/mL	500 μL
Saline	n/a	8.6 mL
Total	n/a	10 mL

Prepare the solution in sterile tubes/vials aseptically in a biosafety cabinet.

**17. Collagenase D solution**

**Table:** 

Reagent	Final concentration	Quantity or Volume
Collagenase D	100 mg/mL	500 mg
ddH_2_O	n/a	5 mL

Prepare the solution in sterile tubes/vials aseptically in a biosafety cabinet. Aliquot at 500 μL/aliquot in sterile tubes and store at -80 °C.

**18. DNase I solution**

**Table:** 

Reagent	Final concentration	Quantity or Volume
DNase I	100 mg/mL	100 mg
ddH_2_O	n/a	1 mL

Prepare the solution in sterile tubes/vials aseptically in a biosafety cabinet. Aliquot at 50 μL/aliquot in sterile tubes and store at -80 °C.

**19. Lung digestion solution**

**Table:** 

Reagent	Final concentration	Quantity or Volume
RPMI 1640 (with HEPES and L-glutamine)		44.45 mL
FBS	10%	5 mL
Collagenase D (100 mg/mL)	1 mg/mL	500 μL
DNase I (100 mg/mL)	0.1 mg/mL	50 μL
Total	n/a	50 mL

Prepare the solution in sterile tubes/vials aseptically in a biosafety cabinet.

**20. Red blood cell (RBC) lysis buffer**

**Table:** 

Reagent	Final concentration	Quantity or Volume
NH_4_Cl	0.15 M	8.29 g
KHCO_3_	0.01 M	1 g
Na_2_-EDTA	0.1 mM	37.2 mg
Deionized water		up to 1,000 mL

Adjust pH to 7.2–7.4; sterilize using a 0.22 μm filter; store at 4 °C.

**21. Flow cytometry staining buffer**

**Table:** 

Reagent	Final concentration	Quantity or Volume
BSA	1%	0.5 g
PBS		50 mL

**22. Culture medium**

**Table:** 

Reagent	Final concentration	Quantity or Volume
FBS	10%	10 mL
Penicillin-Streptomycin (10,000 U/mL)	100 U/mL	1 mL
RPMI 1640 medium		89 mL

**23. PMA stock solution**

**Table:** 

Reagent	Final concentration	Quantity or Volume
PMA	100 μg/mL	0.1 mg
DMSO		1 mL

Prepare the solution in sterile tubes/vials aseptically in a biosafety cabinet. Aliquot at 50 μL/aliquot in sterile tubes and store at -80 °C.

**24. Ionomycin stock solution**

**Table:** 

Reagent	Final concentration	Quantity or Volume
Ionomycin	1 mM	1 mg
RPMI 1640 medium		1.34 mL

Prepare the solution in sterile tubes/vials aseptically in a biosafety cabinet. Aliquot at 50 μL/aliquot in sterile tubes and store at -80 °C. Protect from light.

**25. 1× permeabilization buffer**

**Table:** 

Reagent	Final concentration	Quantity or Volume
10× permeabilization buffer	1×	10 mL
Distilled water		90 mL

**Laboratory supplies**

1. Weighing boats/papers (VWR, catalog numbers: 12578-121, 12578-165, 12578-201, 10770-442, 10770-448, 10770-450)

2. Glass bottles with cap for solution storage (50, 500, and 1,000 mL) (Corning, catalog numbers: 139550, 1395500, 13951L)

3. Microcentrifuge tubes, 1.5 mL (VWR, catalog number: 87003-294)

4. Microcentrifuge tubes, 2.0 mL (VWR, catalog number: 87003-298)

5. Conical centrifuge tubes, 15 mL (VWR, catalog number: 89039-666)

6. Conical centrifuge tubes, 50 mL (VWR, catalog number: 89039-658)

7. Round-bottom culture tubes for bacteria growth (Corning, catalog number: 352059)

8. Flasks for bacteria culture (Corning, catalog number: 5320-250, 5320-500, 5320-1L)

9. Round-bottom polystyrene test tubes, 5 mL (Corning, catalog number: 352008)

10. Pipette tips (10, 200, and 1,000 μL) (Thomas Scientific, catalog numbers: 1158U35, 1158U56, 1158U31, 1159M43, 1159M40, 1159M42)

11. Serological pipettes, sterile (5, 10, and 25 mL) (Fisher Scientific, catalog number: 13-678-11D, 13-678-11E, 13-678-11)

12. Petri dishes (Fisher Scientific, catalog number: FB0875712)

13. Ice

14. Dry ice

15. Liquid nitrogen

16. 70% alcohol (Medline, catalog number: MDS098016)

17. Sterile gauze (Medline, catalog number: NON21420)

18. Sterile syringe filters, 0.22 μm pore size (Millipore Millex, catalog number: SLGP033RS)

19. Syringes (1, 5, and 10 mL) (Becton, Dickinson and Company, catalog numbers: 309659, 309647, 303134)

20. VetriJec insulin syringe U-100 (0.3 cc, 0.5 cc) (AmerisourceBergen MWI Animal Health, catalog numbers: 601086, 510107)

21. Needles (27 G, 30 G) (Becton, Dickinson and Company, catalog numbers: 305109, 305106)

22. Heating pad (Sunbeam, catalog number: 731-500) or small animal heat lamp (Morganville Scientific, catalog number: HL0100)

23. Blood collection system MiniCollect^TM^ capillary tubes (Greiner Bio-One, catalog number: 450476)

24. Sharp-pointed dissecting scissors (Fisher Scientific, catalog number: 08-940, 08-935)

25. Fine-tipped forceps (Roboz, catalog number: 08-940, 08-935)

26. Microcalipers (Walter Stern, catalog number: 602B)

27. 6-well tissue culture plate (Corning, catalog number: 353046)

## Equipment

1. Freezer (-80 °C) (Thermo Fisher Scientific, model: 989-86C)

2. Freezer (-20 °C) (Bio Medical Solutions, SCGP26OW1AF)

3. Refrigerator (2–8 °C) (True Refrigeration, model: GDM-47)

4. Biosafety cabinet (Nuaire, model: LabGard, Class II, Type A2)

5. Pipettes (P10, P100, P200, P1000) (Eppendorf, catalog numbers: 3123000020, 3123000047, 3123000055, 3123000063)

6. Portable Pipet-Aid^TM^ XP Pipet Controller (Drummond, catalog number: 4000101)

7. Centrifuge (Thermo Fisher Scientific, model: Sorvall X4R Pro-MD)

8. Microcentrifuges (Eppendorf, models: 5425/5425 R)

9. Microbiological incubator (Thermo Fisher Scientific, catalog number: 51028067)

10. Shaker (Eppendorf, model: New Brunswick Innova 42, catalog number: M1335-0000)

11. Balances (Ohaus, model: Adventurer Pro AV213; Mettler, model: PE160; Sartorius, model: CP225D; Ohaus, model: STX223)

12. Dissecting microscope (Leica Microsystems, model: S9i)

13. NanoDrop (Thermo Fisher Scientific, model: Nanodrop One)

14. Flow cytometer (BD Biosciences, model: LSRFortessa I)

15. Thermal cycler for qPCR (Thermo Fisher Scientific, model: ProFlex)

16. Microtome (Leica, model: HistoCore BIOCUT)

17. Cryostat for tissue sectioning (Leica, model: CM1950)

18. Microscope for immunohistological/immunofluorescence analysis (Leica, model: DMi8)

19. Gel electrophoresis apparatus (Bio-Rad, model: Sub-Cell GT)

20. CO_2_ incubator (Thermo Fisher Scientific, model: Heracell 150i)

21. Gel imager (Bio-Rad, model: ChemiDoc imaging system)

## Software and datasets

1. FlowJo for flow cytometry analysis (FlowJo, Version 10.7)

2. GraphPad Prism for statistical analysis (GraphPad, Version 8)

## Procedure

**A. Lung tumor induction**

*Note: Use one-way analysis of variance (ANOVA) power analysis to determine the minimum sample size, which will be calculated based on the effect size (usually expressed in standard deviation units), the number of groups, the power level, and the statistical significance you want to achieve.*

1. Inject 6–8-week-old mice intraperitoneally (IP) with urethane (1 mg/g body weight) weekly for 6 weeks. Weigh each mouse. Record body weight and calculate the volume of the urethane solution to be injected. For 100 mg/mL urethane solution (see Recipe 3), the injection volume will be 0.1 mL per 10 g of body weight.

2. Disinfect the top of the multidose urethane solution container, including the cap and the cap surroundings, with 70% alcohol and gauze.

3. Draw up, into the syringe and needle, the amount of urethane solution to be administered.

4. Gently restrain the mouse by grasping the scruff of the neck.

5. With the bevel facing up, insert the needle at a 30–45° angle. Pull back on the plunger to ensure negative pressure (a resistance to the pull force) prior to injecting. If there is no negative pressure, the needle tip is possibly within a blood vessel, and blood may come out and be visible in the needle/syringe. If there is negative pressure, proceed with the injection. Inject into the lower right or left quadrant of the abdomen, avoiding the midline to prevent organ injury. Slowly inject the solution and carefully withdraw the needle.

6. Monitor the mouse for any complications. Urethane treatment will also induce anesthesia in mice. After administration of urethane, move the mice to a dry, clean area and monitor regularly (at least every 15 min). To minimize hypothermia, keep the mice warm with a circulating water blanket, warm water bottle, blankets, blue diaper pads, heat discs, a glove filled with warm water, or other methods. Return the mice to their routine housing only after they have recovered from anesthesia (i.e., the animal can maintain itself in sternal recumbency and is fully ambulatory. Sternal recumbency means that the animal can hold itself upright with its chest resting on the ground, which indicates that the animal has regained consciousness and has sufficient muscle control to support its weight). Observe the mice daily, and note any signs of distress (e.g., decreased activity, rough coat, labored breathing). Record weight, body condition, and behavior at least weekly.

**Critical:**

1. Perform injection consistently to minimize variability:

• Restrain the mouse gently but firmly to prevent movement during injection.

• Use the same type and gauge of needles (e.g., 30 G) for all injections.

• Insert the needle at a 30–45° angle with the bevel facing up.

• Inject the solution slowly and steadily to avoid tissue damage or reflux (the injected solution leaking back out of the injection site).

• Practice with saline injections if you are new to the procedure to ensure reproducibility.

2. Minimize animal stress by handling mice gently and calmly at all times:

• Perform all injections and procedures in a quiet environment to reduce anxiety.

• Use appropriate restraint techniques to avoid struggling or injury and limit the duration of restraint to the minimum necessary.

• Allow animals to recover in a warm, comfortable area after procedures, and monitor them closely for signs of distress (such as vocalization, rapid breathing, or abnormal behavior).

• If excessive stress or distress is observed, pause the procedure and allow the animal to recover before continuing.

**B. Plasmid DNA preparation**

1. Transformation of competent cells

a. Thaw chemically competent *E. coli* on ice for 10 min.

b. Add 1–5 ng of pCMV-myc-PDLIM2 or control vector pCMV plasmid to 50 μL of competent cells.

c. Gently flick the tube to mix and incubate on ice for 30 min.

d. Perform heat shock at 42 °C for 45 s, then immediately place on ice for 2 min.

e. Add 450 μL of SOC or LB medium to the tube and incubate at 37 °C for 1 h with shaking (225 rpm).

f. Spread 100–200 μL of the culture onto an LB agar plate with ampicillin (100 μg/mL).

g. Incubate overnight at 37 °C.

2. Plasmid amplification and purification

a. Pick a single colony from the plate and inoculate a starter culture of 2–5 mL of LB medium with ampicillin (100 μg/mL). Incubate for approximately 8 h at 37 °C with vigorous shaking (approximately 300 rpm). Use a tube or flask with a volume of at least 4 times the volume of the culture.

b. Dilute the starter culture 1/500 to 1/1,000 into LB medium with ampicillin (100 μg/mL). Use a flask or vessel with a volume of at least 4 times the volume of the culture. Inoculate 100 mL of medium with 100–200 μL of starter culture.

c. Grow at 37 °C for 12–16 h with vigorous shaking (approximately 300 rpm). The culture should reach a cell density of approximately 3–4 × 10^9^ cells per milliliter, which typically corresponds to a pellet wet weight of approximately 3 g/L medium.

d. Extract plasmid DNA using an EndoFree Plasmid Maxi kit following the manufacturer’s protocol.

e. Quantify DNA concentration using NanoDrop.

f. Confirm plasmid presence and integrity using restriction digestion or agarose gel electrophoresis.

**Critical:**

1. Use the correct amount of plasmid to optimize transformation efficiency.

2. Precise timing of heat shock is crucial for cell viability and plasmid uptake.

3. Ensure proper antibiotic concentration to select for transformed cells.

4. Use an EndoFree kit to minimize endotoxin contamination for in vivo applications.

5. Verify plasmid identity before proceeding.

6. Accurate quantification is essential for reproducible nanoPlasmid preparation.

**C. Preparation of NanoPDLIM2**

NanoPDLIM2 preparation for intravenous injection per mouse per time is described below. Scale up according to the number of mice to be administered plus 10% extra to offset the loss in the procedures.

1. Prepare DNA solution

a. Dilute 25 μg of plasmid DNA in 50 μL of 10% glucose solution.

b. Add sterile water to reach 100 μL.

c. Vortex gently and spin down briefly (no more than 1 min).

2. Prepare in vivo-jetPEI solution

a. Dilute 4 μL of in vivo-jetPEI in 50 μL of 10% glucose solution.

b. Add sterile water to reach 100 μL.

c. Vortex gently and spin down briefly (no more than 1 min).

3. Nanoparticle complex formation

a. Add the diluted in vivo-jetPEI to the diluted DNA solution at once.

b. Vortex briefly and spin down.

c. Let the mixture sit at room temperature for 15 min to allow complex formation; then, perform injection.

**Critical:**

1. Use high-quality plasmid preparation. Ensure they contain neither salt, RNA, protein, or endotoxin. It is best to use DNA dissolved in water and with an OD_260/280_ ratio greater than 1.8.

2. Rapid mixing of the DNA solution with the in vivo-jetPEI solution is important for proper nanoparticle complex formation.

**D. Treatment administration**

**Figure 1.**
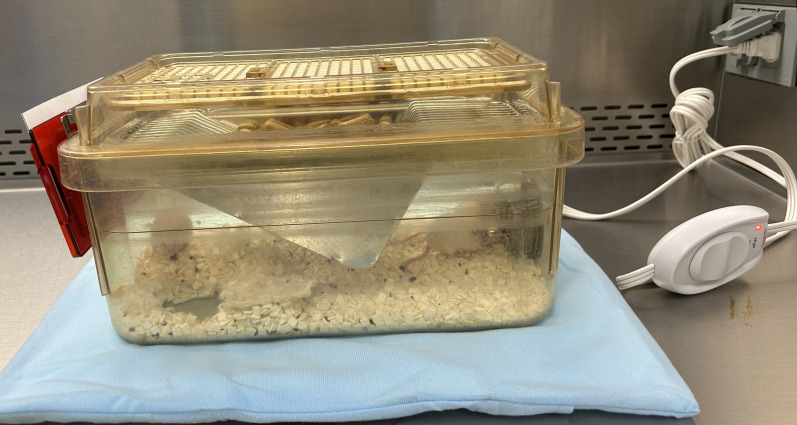
Mouse vein dilation induction with a heating pad

1. Inject mice intravenously with nanoPlasmid (NanoPDLIM2 or NanoVector, 200 μL per mouse) weekly for 6 weeks.

a. Place the mouse in a restrainer.

b. Warm the tail using a heating pad or heat lamp for 2–5 min to dilate the veins (Figure 1).

c. Load 200 μL of nanoPDLIM2 solution into a 30 G insulin syringe.

d. Remove air bubbles by gently tapping the syringe and expelling excess liquid.

e. Use the lateral tail veins (visible on both sides of the tail).

f. Disinfect the injection site with an alcohol pad.

g. Hold the syringe at a 10–15° angle.

h. Insert the needle into the vein and aspirate slightly. If blood enters the syringe, proceed with injection.

i. Slowly inject 200 μL over 5–10 s to avoid vein rupture.

j. If resistance is felt or swelling occurs, stop immediately and try another vein.

k. Gently press the injection site with sterile gauze to prevent bleeding.

l. Return the mouse to its cage and monitor for 15–30 min for adverse reactions.

2. IP-inject mice once a week with carboplatin injection solution (3 mg/mL) and paclitaxel injection solution (1.5 mg/mL) or control vehicles (control injection solutions with everything the same as the carboplatin/paclitaxel injection solutions except that PBS replaces carboplatin/paclitaxel stock solutions) at 100 μL/10 g of body weight for the dose of carboplatin at 30 mg/kg body weight and paclitaxel at 15 mg/kg body weight each time. To reduce the inhibition of DMSO on carboplatin’s activity, IP-inject all mice with carboplatin first, and then paclitaxel.

3. IP-inject mice 3 times a week with 200 μL of anti-PD-1 or control IgG antibody (1 μg/μL) for a dose of 200 μg/mouse each time.

4. Repeat treatments weekly for 6 weeks.

5. To assess treatment-induced toxicity, monitor and record mouse health status and behavior at least daily. Record mouse weight at least once per week. If a mouse shows severe signs of pain and distress, such as severe difficulty in breathing or moving, significant weight loss (20% or greater loss compared with before the start of treatment), moribund states of arousal, or a body condition score (BCS) that reaches < 2/5, euthanize the mouse. Collect the blood and analyze cytokine/chemokine levels and hepatic enzymes in serum as described previously [45]. Examine the various organs for signs of abnormality. Collect the tissues and perform histological examination for pathological changes as we described before [7,8,10,21,26, 28,29,31,36,37,46–50].

*Note: For intravenous injection into the lateral tail veins, start from the distal part of the tail (away from the body) and move proximally (toward the base of the tail, closer to the body) in case multiple attempts are needed. After one unsuccessful attempt, inject at a more proximal location to avoid any potentially clogged or damaged sites, rendering the injection unsuccessful again. Ensure the needle bevel is facing up. Carefully insert the needle parallel to the vein and inject the solution slowly to minimize trauma.*

**Critical:**

1. Randomly assign animals to different treatment groups to minimize bias and improve the validity of the experiment. Randomization helps to ensure that any other factors (e.g., environment, handling) are distributed equally across the groups, minimizing the chance that these factors could influence the results, making it more likely that any differences between groups are due to the treatment itself, rather than pre-existing variations among the animals.

2. Ensure slow and steady injection to avoid injury. Observe the mouse for any signs of distress during and immediately after injection.

**E. Tissue dissection and processing**

1. Euthanize mice 6 weeks post last urethane administration. Before mouse euthanasia, collect blood by cardiac puncture, retro-orbital sinus, or tail vein puncture, in anticoagulant-containing tubes for blood cell and plasma analysis, or in non-anticoagulant-containing tubes for serum analysis. Blood collection via cardiac puncture or retro-orbital sinus is a terminal procedure that needs to be performed under mouse general anesthesia. To induce mouse anesthesia, weigh the mouse. Record body weight and IP-inject the anesthesia solution (see Recipe 16) at 0.1 mL per 10 g of body weight. Proceed to blood collection when the mouse has no response to a toe pinch, which indicates that anesthesia is sufficient. After blood collection, euthanize the mouse by manual cervical dislocation.

2. Expose the thoracic cavity by making an incision from the abdomen to the sternum.

3. Carefully cut the ribcage to open the chest and expose the lungs.

4. If blood clearance is needed, perform cardiac perfusion with PBS before lung removal.

5. Carefully detach the lungs from the trachea and place them in a Petri dish with PBS.

6. Under a dissecting microscope, examine the lung for tumors, which appear as white or opaque nodules on the lung surface. Measure tumor diameter with a microcaliper. Record tumor number, size, and distribution.

7. Carefully examine all the other organs besides the lung, including the liver, spleen, kidney, stomach, intestine, colon, thymus, heart, lymph nodes, etc. Record and take pictures/videos of any abnormalities observed. Collect tissue samples, particularly lung and lung tumors, for various analyses. For tissue pieces collected for flow cytometry and/or single-cell RNA sequencing (scRNAseq), immediately proceed to tissue dissociation, single-cell isolation, and flow cytometry staining or scRNAseq procedures. Flash freeze tissue pieces for later molecular analysis by qPCR, RNA sequencing, western blot, etc. Place tissue pieces in NBF for later immunohistochemistry (IHC)/immunofluorescence (IF) staining and analysis. Embed tissue pieces in OCT compound for later frozen sectioning, IF staining, and analysis.

*Note: Handle tissues carefully to avoid damage.*

**Critical:** Proper tissue processing is essential for accurate downstream analysis.

**F. Single cell isolation for flow cytometry**

1. Place the lung lobes in the cap of a tube on ice. Using sterile scissors, mince the lobes into small pieces. Keep the cap on ice while processing all the samples.

2. Transfer the minced lung tissue into a tube containing 2 mL of prewarmed digestion buffer. Mix gently to ensure all tissue fragments are submerged.

3. Incubate the tube at 37 °C for 45 min with gentle shaking.

4. Transfer the digestion mixture onto a 70 μm cell strainer placed over a 50 mL conical tube.

5. Using the rubber plunger of a syringe, gently press the lung tissue fragments through the strainer until no visible chunks remain.

6. Rinse the strainer and syringe with 5 mL of flow cytometry staining buffer to collect the remaining cells.

7. Centrifuge at 400× *g* for 10 min at room temperature.

8. Carefully aspirate and discard the supernatant without disturbing the cell pellet.

9. Resuspend the pellet in 1–2 mL of RBC lysis buffer.

10. Incubate for 5 min at room temperature.

11. Neutralize the RBC lysis buffer by adding 10 times the volume of flow cytometry staining buffer.

12. Centrifuge again at 400× *g* for 5 min at room temperature.

13. If intracellular cytokine flow cytometry analysis will be performed, resuspend the cell pellet in culture medium. Otherwise, resuspend the cell pellet in flow cytometry staining buffer, count the cells, and proceed to flow cytometry staining.

**Critical:** Optimize digestion time and enzyme concentration to maximize cell yield and viability.

**G. Preparation for intracellular cytokine staining**

1. Add the cells in 6-well plates to culture.

2. Add to the cell culture the following reagents to the final concentration indicated: PMA (50 ng/mL), ionomycin (1 μM), brefeldin A [BFA, 1× (3 μg/mL)], and monensin [1× (2 μM)].

3. Culture the cells in 5% CO_2_ in a humidified CO_2_ incubator at 37 °C for 4 h.

4. Collect the cells into centrifuge tubes and centrifuge at 400× *g* for 5 min at room temperature.

5. Resuspend the cell pellet in flow cytometry staining buffer, count the cells, and proceed to flow cytometry staining.

*Notes:*

*1. PMA and ionomycin are used to stimulate the production of cytokines, such as interferons (IFNs).*

*2. As cytokines are secreted proteins, BFA and monensin, which can inhibit protein transport from the endoplasmic reticulum to the Golgi apparatus, are used to inhibit secretion of the cytokines out of the cells and make them accumulate inside the cells for flow cytometry analysis.*

**H. Flow cytometry staining and analysis**

1. Aliquot 1–2 × 10^6^ cells per 5 mL tube.

2. Centrifuge at 400× *g* for 5 min at 4 °C to pellet the cells and discard the supernatant.

3. Resuspend the cell pellet in 50 μL of anti-CD16/CD32 solution per sample (Fc block Ab diluted 1:100 in flow cytometry staining buffer) and incubate for 5 min at room temperature.

4. Prepare a master mix of fluorochrome-conjugated antibodies to cell surface antigens in flow cytometry staining buffer.

5. Add 50 μL of the antibody mix per sample tube and incubate for 30 min at room temperature in the dark.

*Note: Protect from light for all subsequent steps.*

6. Wash twice with 1 mL of flow cytometry staining buffer and centrifuge at 400× *g* for 5 min at 4 °C. Discard the supernatant.

7. Resuspend the pelleted cells in 200 μL of flow cytometry staining buffer for flow cytometry analysis or proceed to intracellular staining.

8. Intracellular staining:

a. Fix the cells by adding 100 μL of IC fixation buffer and pulse vortex to mix.

b. Incubate for 20–60 min at room temperature in the dark.

c. Wash twice with 2 mL of 1× permeabilization buffer and centrifuge at 400× *g* for 5 min at room temperature. Discard the supernatant.

d. Resuspend the cell pellet in 100 μL of 1× permeabilization buffer. Add conjugated primary antibodies for intracellular antigen(s) to cells and incubate for 20–60 min at room temperature in the dark.

e. Wash twice with 2 mL of 1× permeabilization buffer and centrifuge at 400× *g* for 5 min at room temperature. Discard the supernatant.

f. Resuspend stained cells in an appropriate volume of flow cytometry staining buffer for flow cytometry analysis.

g. Analyze the samples on a flow cytometer such as BD Biosciences LSRFortessa I.

**Critical:**

1. Use compensation beads for single-color controls.

2. Set gates based on unstained and fluorescence minus one (FMO) controls.

## Data analysis

**A. Tumor evaluation**

**Figure 2.**
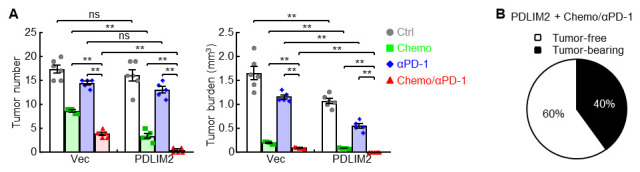
Combination of PDLIM2 nanotherapy, chemotherapy, and immunotherapy shows great efficacy in lung cancer treatment. (A) Tumor examination showing the strongest effect of the three-combination therapy on reducing tumor number and tumor burden (n ≥ 5). Student’s t-test was performed (two-tailed, unpaired), and data represent means ± SEM. *p < 0.05; **p < 0.01; ns, not statistically significant. (B) Tumor examination showing complete remission of all lung tumors in 60% of mice by the combination of the three therapies. Reprinted/adapted from Sun et al. [10]. © The Authors, some rights reserved; exclusive licensee, eLife Sciences Publications Ltd. Distributed under a Creative Commons Attribution license Attribution 4.0 International (CC BY 4.0).

Upon mouse euthanasia, under a dissecting microscope, count lung tumors, which appear as white or opaque nodules on the lung surface. Measure tumor diameters with a microcaliper. Calculate tumor burden for each mouse as the sum of the volumes (Vs) of each tumor [V = (4/3)π(D/2)^3^ for a round tumor with diameter D]. Compare tumor number, size distribution, and burden between different treatment groups. In addition, perform histological evaluation of lung tumors on hematoxylin and eosin (H&E)-stained lung tissue sections. All three combination of chemo (carboplatin/paclitaxel)/immuno (αPD-1)/NanoPDLIM2 treatment showed the strongest effect on reducing tumor burden and numbers, causing complete remission of lung tumors in 60% of mice and substantial lung tumor reduction in the remaining mice in comparison to those treated with two combined therapies (Figure 2), demonstrating superior efficacy of the NanoPDLIM2-based combination therapy in treating lung cancer in mice.

**B. NanoPlasmid delivery and delivered gene expression analysis**

**Figure 3.**
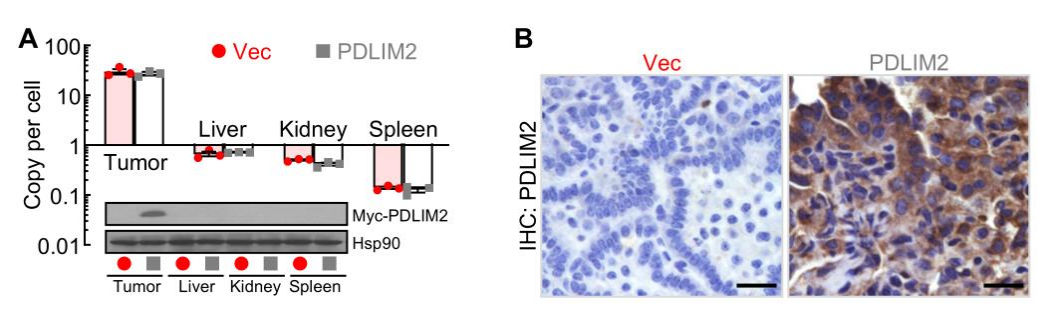
PDLIM2 nanotherapy reconstitutes PDLIM2 specifically in lung tumors. (A) PCR and immunoblotting (IB) assays showing lung tumor-specific plasmids delivery and PDLIM2 expression by PDLIM2 nanotherapy (n = 3). Data represent means ± SEM. (B) Immunohistochemistry (IHC) staining showing high PDLIM2 re-expression in lung tumors after PDLIM2 nanotherapy. Scale bar: 20 μm. Reprinted/adapted from Sun et al. [10]. © The Authors, some rights reserved; exclusive licensee, eLife Sciences Publications Ltd. Distributed under a Creative Commons Attribution license Attribution 4.0 International (CC BY 4.0).

Perform PCR as we described before [10,46], using the primers shown in Table 2, to quantify the delivered plasmids in various tissues at different time points post-NanoPlasmid administration. Perform immunoblotting (IB) and/or immunostaining analysis as described before [46,47] to examine the delivered ectopic PDLIM2 protein expression in various tissues. Our analysis demonstrated lung tumor–specific plasmid delivery and PDLIM2 reconstitution by PDLIM2 nanotherapy (Figure 3).

**Table 2.** Primers for NanoPlasmid delivery and delivered gene expression analysis

Gene	Species	Accession number	Forward (5′ to 3′)	Reverse (5′ to 3′)	Usage
*Amp-R*	Plasmid		AACTTTATCCGCCTCCAT	GAAGCCATACCAAACGAC	Plasmid DNA quantification
*Lyz2*	mouse	NC_000076.6	AGCAGGCATGCTTTCTCTAGTC	GCGATTAGCTGGAGCCATCA	

**C. Toxicity examination**

**Figure 4.**
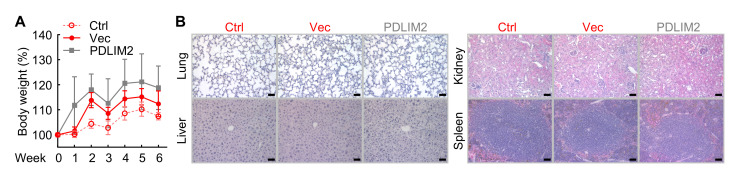
PDLIM2 nanotherapy causes no obvious toxicity in mice. (A) No significant effect of PDLIM2 nanotherapy on the body weight of mice treated with anti-PD-1 and chemotherapeutic drugs (n = 5). Data represent means ± SEM. (B) H&E staining showing comparable toxicity in lung, liver, kidney, and spleen between the Vec and PDLIM2 groups in the context of combinational chemotherapy and PD-1 blockade immunotherapy (n = 5). Scale bar: 50 μm. Reprinted/adapted from Sun et al. [10]. © The Authors, some rights reserved; exclusive licensee, eLife Sciences Publications Ltd. Distributed under a Creative Commons Attribution license Attribution 4.0 International (CC BY 4.0).

Monitor and compare body weight change and other health and behavior changes over time between treatment groups. In addition, perform histological examination of various tissues for pathological changes as described before [7,8,10,21,26,28,29,31,36,37,46–50]. Our analysis indicated that PDLIM2 nanotherapy caused no obvious toxicity in mice (Figure 4).

**D. Immune and molecular analysis**

**Figure 5.**
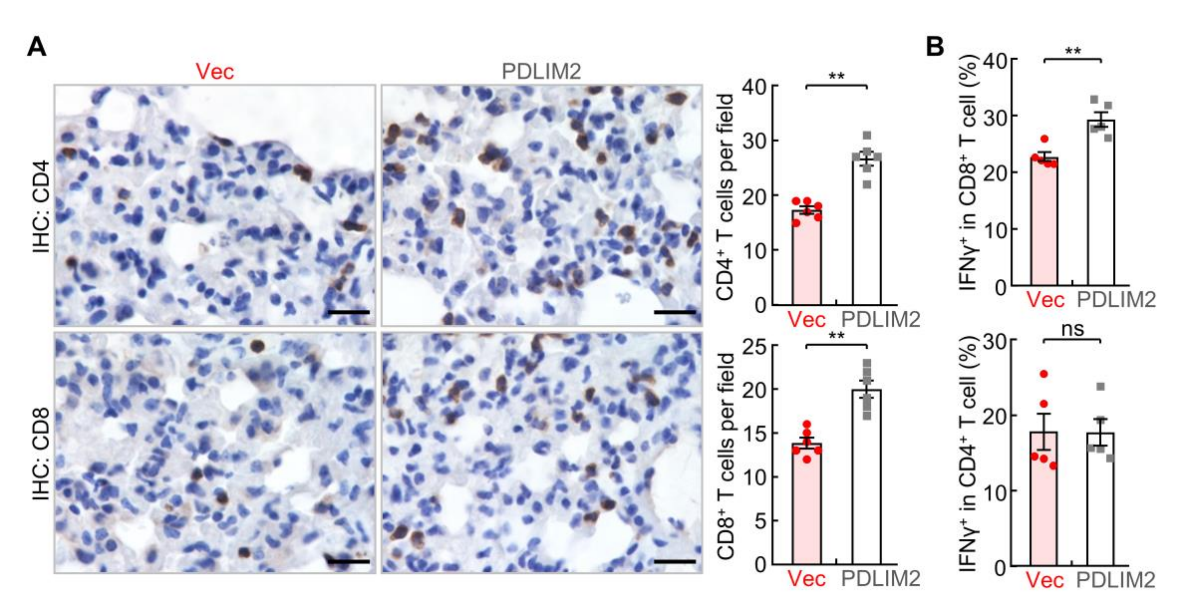
PDLIM2 nanotherapy increases tumor-infiltrating lymphocytes (TILs) and cytotoxic T-cell activation. (A) IHC staining showing increased TILs by PDLIM2 nanotherapy in mice treated with anti-PD-1 and chemotherapeutic drugs (n = 6). Scale bar, 20 μm. (B) Flow cytometry analysis showing increased production of IFNγ in lung CD8^+^ T cells, indicating increased CD8^+^ T-cell activation, by PDLIM2 nanotherapy in mice treated with anti-PD-1 and chemotherapeutic drugs (n = 5). Student’s t-test was performed (two-tailed, unpaired), and data represent means ± SEM. **p < 0.01; ns, not statistically significant.

Perform immune and molecular analysis to elucidate the cellular and molecular mechanisms underlying the treatment efficacy. Examine immune cell composition and activation status in the lung, lung tumors, and other tissues by flow cytometry and IHC/IF analysis as described in the procedure and in previous publications [7,8,10,21,26, 28,29,31,36,37,46–50]. Our analysis indicates that PDLIM2 nanotherapy increases lymphocyte tumor infiltration and cytotoxic T-cell activation in the lung (Figure 5). These may contribute to the anti-tumor efficacy of the PDLIM2 nanotherapy.

## Validation of protocol

This protocol or parts of it has been used and validated in the following research article(s):

• Sun et al. [7]. Dual but not single PD-1 or TIM-3 blockade enhances oncolytic virotherapy in refractory lung cancer. *J. Immunother. Cancer.* (Figures 1G–1I, 2A, 2D–2F, 3D–3H, and 4–6)

• Sun et al. [8]. Causative role of PDLIM2 epigenetic repression in lung cancer and therapeutic resistance. *Nature Communications*. (Figures 4a, 4h–4l, 5b, 5d, 5e, 5h, 6a, 7c, 7f, 7h, and 7k–7m)

• Sun et al. [10]. Improving PD-1 blockade plus chemotherapy for complete remission of lung cancer by nanoPDLIM2. *eLife*. 12: RP89638. (Figures 2–6)

• Li et al. [21]. PDLIM2 repression by ROS in alveolar macrophages promotes lung tumorigenesis. *JCI Insight.* (Figures 1A–G, 1I, 2–4, 5A–C, and 6G–K)

• Li et al. [26]. NF-kappaB RelA renders tumor-associated macrophages resistant to and capable of directly suppressing CD8(+) T cells for tumor promotion. *Oncoimmunology* 7, e1435250. (Figures 1A and B, 2, 3, 4A–D, 5D, G, and H, and 6A, E, and F)

• Zhou et al. [28]. Differential roles of STAT3 in the initiation and growth of lung cancer. *Oncogene*. (Figures 1–5)

• Zhou et al. [31]. Myeloid STAT3 Promotes Lung Tumorigenesis by Transforming Tumor Immunosurveillance into Tumor-Promoting Inflammation. *Cancer Immunol Res.* (Figures 1A, 1B, 2–4, and 7)

• Sun et al. [36]. NF-kappaB1 p105 suppresses lung tumorigenesis through the Tpl2 kinase but independently of its NF-kappaB function. *Oncogene* 35, 2299–2310. (Figures 2, 3, 4a, 5b, and 7)

• Sun et al. [37]. Critical and distinct roles of cell type-specific NF-κB2 in lung cancer. *JCI Insight*. 9, e164188. (Figures 1, 2A, 3, 4A and B, 5A and B, 6, and 7A–C)

## General notes and troubleshooting

**General notes**

1. Reagent quality

All reagents were of molecular biology grade or higher, and sterile technique was rigorously maintained throughout all procedures to minimize the risk of contamination. Particularly stringent aseptic practices were observed during plasmid preparation, nanoparticle formulation, solution preparation, and during all handling of cells and tissues. Where possible, critical steps were conducted within a certified biological safety cabinet to further safeguard experimental integrity.

2. Reagent preparation

All solutions, including those for nanoPDLIM2 complexes, chemotherapeutic agents, and antibody preparations, were prepared by carefully verifying concentrations and volumes against calculated values. Small deviations in reagent preparation were recognized to have profound effects on experimental outcome; thus, all calculations were independently cross-checked. Reagents were confirmed to be within expiration dates and were stored under manufacturer-recommended conditions (e.g., -80 °C for plasmids, antibodies, and chemotherapeutics; 4 °C for most aqueous buffers and media). Solutions were visually inspected prior to use, and those with any signs of precipitation, turbidity, or discoloration were discarded and remade using fresh, sterile stock components. Where appropriate, buffers and working solutions were sterilized by filtration through 0.22 μm membranes.

3. Documentation

Comprehensive records were maintained for all reagents and samples, including supplier and lot numbers, preparation dates, concentrations, and storage conditions. Each batch of plasmid DNA, nanoparticle formulations, antibody stocks, and major buffer solutions was logged in a dedicated laboratory inventory system. This thorough documentation ensures traceability and allows for retrospective analysis in the event of batch-to-batch variability or experimental discrepancies.

4. Animal welfare

Experiments involving animals were conducted in compliance with IACUC-approved protocols. Mice were inspected at least once daily for signs of distress, toxicity, or aberrant behavior, particularly following tumor induction and drug administration, with all observations and interventions recorded. All animal housing and procedures conformed to institutional and national standards for laboratory animal care.

5. Batch consistency

Whenever feasible, all critical reagents (including nanoPDLIM2 complexes and injection solutions) were prepared in single, uniform batches to ensure reproducibility and minimize cross-sample variability. Aliquots from the same batch were used across all replicate experiments and treatment groups, and batch stability was monitored by storage under controlled conditions and, where applicable, functional or physiochemical verification.

6. Experimental controls

Each experiment included appropriate negative controls (such as vehicle controls for chemotherapeutic drugs and PD-1 and/or empty vector) and positive controls (known effective treatments or standards-of-care) in both in vivo and in vitro assays. Control groups were analyzed in parallel with experimental groups to validate assay specificity and overall assay performance.

7. Data recording and management

All experimental procedures, observations, and endpoint analyses were recorded immediately using standardized data collection forms and/or secure electronic laboratory notebooks. Protocol deviations, adverse events, and unexpected findings were documented contemporaneously to ensure data transparency and facilitate reproducibility.

8. Laboratory safety

All hazardous chemicals (including, but not limited to, urethane and chemotherapeutic agents) were handled in accordance with institutional and governmental safety protocols. Laboratory personnel wore appropriate personal protective equipment at all times, and work with volatile or toxic agents was performed in certified chemical fume hoods. Hazardous waste was disposed of via approved institutional procedures. Emergency equipment (e.g., eyewash stations, safety showers, spill response kits) was available and personnel was trained in relevant emergency protocols.

**Troubleshooting**

**Problem 1: Low plasmid yield.**

Possible causes: Inefficient bacterial growth, compromised lysis during plasmid extraction, or use of outdated reagents may result in reduced plasmid DNA recovery.

Solutions: Ensure optimal bacterial culture conditions (appropriate temperature, aeration), optimize the lysis step during plasmid extraction (e.g., adjust incubation times, reagent volumes), and use fresh competent cells and high-quality LB/ampicillin media.

**Problem 2: Low transfection efficiency.**

Possible causes: Poor plasmid integrity or an improper ratio of PEI to DNA during nanoparticle formulation can hinder efficient transfection.

Solutions: Confirm plasmid integrity by agarose gel electrophoresis to rule out degradation or structural issues. Optimize the PEI:DNA ratio to ensure efficient complex formation and cellular uptake.

**Problem 3: Mortality occurs post-injection.**

Possible causes: Incorrect dosing calculations, improper injection technique, or pre-existing stress/dehydration in animals can lead to post-injection mortality.

Solutions: Thoroughly review dosing calculations and confirm accurate drug concentrations. Validate the injection technique to minimize tissue damage and ensure proper drug delivery. Ensure animals are well-hydrated and minimize stress before and during procedures.

**Problem 4: Inconsistent tumor induction.**

Possible causes: Inaccurate urethane dosing due to improper preparation or pipette calibration or inconsistent injection techniques can lead to variability in tumor induction.

Solutions: Prepare urethane solution fresh on the day of use to minimize degradation. Calibrate pipettes regularly to ensure accurate dosing. Use a consistent injection technique to deliver the urethane solution properly.

**Problem 5: Poor therapeutic response.**

Possible causes: Drug degradation, improper formulation, incorrect dosing schedule, or inappropriate injection route can compromise the therapeutic efficacy of carboplatin, paclitaxel, and/or anti-PD-1 antibody. For immune checkpoint blockade, antibody degradation may occur from repeated freeze-thaw cycles.

Solutions: Verify the activity and proper formulation of all drugs. Confirm the correct dosing schedule and injection route for each agent. Avoid repeated freeze-thaw cycles of the anti-PD-1 antibody to prevent degradation.
